# Corrigendum to “A systematic review on the impact of sub‐epidermal moisture assessments on pressure ulcer/injury care delivery pathways”

**DOI:** 10.1111/iwj.14951

**Published:** 2024-06-25

Avsar P, Patton D, Cuddigan J, Moore Z. A systematic review on the impact of sub‐epidermal moisture assessments on pressure ulcer/injury care delivery pathways. Int Wound J. 2024;21(6):e14928. doi:10.1111/iwj.14928.

The PU incidence for the usual care group was incorrectly stated as 25%. It should be 2.5% (401/15790).

There is a statistically significant difference in visual PI/PU development in favour of the use of care pathways based on SEM assessments (PU incidence 1%, 27/2661, care pathway group, versus 2.5%, 401/15 790 usual care group).”

We apologise for this error.

